# Mesalazine: a novel therapeutic agent for periodontitis via regulation of periodontal microbiota and inhibiting *Porphyromonas gingivalis*

**DOI:** 10.3389/fmicb.2025.1531258

**Published:** 2025-01-22

**Authors:** Yuqi Wang, Jun Ma, Haoyu Wang, Jingzheng Yi, Yuxin Bai, Min Hu, Jiaqing Yan

**Affiliations:** ^1^School and Hospital of Stomatology, Jilin University, Changchun, China; ^2^Jilin Provincial Key Laboratory of Tooth Development and Bone Remodeling, Hospital of Stomatology, Jilin University, Changchun, China; ^3^Western Dental Kids, Fresno, CA, United States

**Keywords:** periodontitis, mesalazine, *Porphyromonas gingivalis*, plaque biofilm, inflammatory bowel disease

## Abstract

**Introduction:**

Periodontitis and inflammatory bowel disease are chronic inflammatory diseases with shared epidemiological, biological, and therapeutic associations. Given the similarities in their pathogenic factors, this study hypothesized that mesalazine, a key treatment agent for inflammatory bowel disease, could also be effective in managing periodontitis.

**Methods:**

The antimicrobial effect of mesalazine on *Porphyromonas gingivalis* was investigated *in vitro*, including observations of morphological changes on the surface of *P. gingivalis*. Additionally, the impact of mesalazine on both the formation and established plaque biofilms was examined. The antimicrobial mechanism was elucidated by assessing the expression of *P. gingivalis* virulence genes and by determining the disruptive effect on *P. gingivalis* cell membranes. An *in vivo* rat model of periodontitis was constructed to evaluate mesalazine’s efficacy and its influence on the periodontal bacterial flora in the context of periodontitis.

**Results and discussion:**

Our results demonstrated that mesalazine concentrations ranging from 0.5 to 2 mg/mL significantly inhibited *P. gingivalis* proliferation over 72 h. Flow cytometry revealed a marked reduction in the number of viable cells following mesalazine treatment. At the nanometer scale, mesalazine induced crumpling and rupture of the *P. gingivalis* surface, compromising cell membrane integrity. Mesalazine not only suppressed the formation of plaque biofilms by *P. gingivalis* and polymicrobial communities but also disrupted pre-existing biofilms. The data also suggested that mesalazine could disrupt the integrity of the *P. gingivalis* cell membrane and inhibit the expression of virulence factors. An animal model of periodontitis in rats was successfully constructed *in vivo*. Mesalazine treatment inhibited alveolar bone resorption, alleviated inflammation of periodontal tissues, and improved the composition of the periodontal flora to a healthier state. This study establishes that mesalazine can treat periodontitis through modulation of the periodontal flora and its anti-inflammatory properties, thus broadening its classical therapeutic applications.

## 1 Introduction

Periodontal disease is a global public health issue that serves as a major cause of tooth loss and is closely related to various systemic diseases, including inflammatory bowel disease (IBD; Slots, 2017). IBD is a non-specific chronic intestinal inflammatory condition (De Souza and Fiocchi, 2016). Recent studies have highlighted that periodontal disease and IBD share common pathogenic mechanisms involving microbiota imbalance and immune response, resulting in mutual pathological promotion and disease progression (Lira-Junior and Figueredo, 2016; Jia et al., 2024). Epidemiological evidence has shown an association between periodontitis and the increased risk of IBD, with reciprocal effects on periodontal health (Kang et al., 2020; Madsen et al., 2023). Biological evidence from clinical studies and animal experiments has shown that IBD and periodontitis are interconnected (Imai et al., 2021). Furthermore, the drugs that are effective for IBD may also be beneficial for preventing and treating periodontitis (Shaw et al., 2016; Lee et al., 2022). Treatment strategies for both conditions focus on reducing inflammation and maintaining microbial balance. Therefore, it is a promising method to treat periodontitis with IBD therapeutics.

Currently, mechanical treatment remains the primary approach for managing periodontitis. However, due to the complex anatomical structures of teeth, simple mechanical intervention often cannot completely eradicate plaque biofilm microorganisms, and adjunctive pharmacological therapy is needed to reduce the number of periodontal pathogenic bacteria in the plaque biofilm. Long-term use of systemic antibiotics could induce gut dysbiosis in the oral microbiota and periodontitis (Yuan et al., 2023). Certain medications used for the treatment of IBD, such as steroids, psoralen, and vitamin D, have shown efficacy in preventing and treating periodontitis by repairing the intestinal inflammatory barrier (Liu et al., 2021; Mishra, 2021). Regardless of the mode of administration, probiotics have shown significant protective effects against alveolar bone loss, which may be due to the changes in the oral and intestinal microbiota (Gatej et al., 2018). Identifying more effective clinical therapeutic agents for periodontitis is essential. The use of IBD therapeutics to treat periodontitis shows promise.

Mesalazine (MSZ), a non-steroidal anti-inflammatory drug, is used as a first-line treatment for IBD and exhibits a wide range of pharmacological characteristics, for instance, antioxidant, anti-inflammatory, antibacterial, antifungal, anticancer, anti-amyloid, gastroprotective, and anti-diverticulosis properties. Although research on the effects of MSZ on microorganisms is limited, studies have demonstrated its role in regulating the gut microbiota (Huang et al., 2022). MSZ has been found to ameliorate colitis and inhibit the expansion of dysbiotic *E. coli* by activating PPAR-*γ* signaling in the intestinal epithelium (Cevallos et al., 2021). Additionally, MSZ upregulated a key operon involved in coordinating stress responses and downregulated bacterial invasiveness by affecting various genes involved in bacterial metabolism. Our previous studies have demonstrated that MSZ can suppress inflammation through specific pathways for the treatment of periodontitis (Wang et al., 2024). However, further investigation is needed to explore its impact on the composition of the bacterial flora in periodontal tissues.

This study aims to investigate the antimicrobial activity and underlying mechanism of MSZ against *Porphyromonas gingivalis* (*P. gingivalis*) by evaluating the effects of MSZ on plaque biofilm formation and pre-formed plaque biofilm, expression of virulence genes, and the morphological structure of the bacterial surface. A rat model of periodontitis was constructed to assess the efficacy of MSZ in periodontitis treatment. Additionally, the impact of MSZ on the composition of the bacterial flora in periodontal tissues was examined using 16S ribosomal RNA (16S rRNA) sequencing.

## 2 Methods

### 2.1 Bacterial strains and animals

The following strains were used: *P. gingivalis* (ATCC 33277), *Fusobacterium nucleatum* (*F. nucleatum*; ATCC 10953), and *Streptococcus sanguinis* (*S. sanguinis*; ATCC 10556). All strains were obtained from the Jilin Provincial Key Laboratory of Tooth Development and Bone Remodeling and were grown in brain–heart infusion (BHI) broth with hemin (5 mg/L) and vitamin K (0.5 mg/L) at 37°C in anaerobic conditions, passaged every 2 days. *P. gingivalis* in the logarithmic growth phase was inoculated into six-well plates (WHB Scientific, Shanghai, China) at a density of 1.0 × 10^8^ CFU/mL and incubated for 2 days in BHI broth with 1% sucrose to establish plaque biofilms. Eighteen 8-week-old healthy SD male rats were purchased from Liaoning Changsheng Biotechnology Co., Ltd., randomly selected, and housed in specific-pathogen-free (SPF) conditions. The room temperature was maintained at 22–24°C, and a 12-h light–dark cycle was maintained. They were allowed free access to tap water and standard chow pellets. Animals were allowed to acclimatize to the environment for 1 week before the experiment. The animal production license number was SCXK (Liao) 2022–0001. The animal experiments were approved by the Ethics Committee on Animal Experimentation of Jilin University, with the approval number SYXK (Ji) 2018–0001.

### 2.2 Growth curves of *Porphyromonas gingivalis* treated with MSZ

MSZ (Aladdin Chemistry, Shanghai, China) was dissolved in BHI broth at a concentration of 2 mg/mL as a stock solution. The concentrations of MSZ (0.5 and 1 mg/mL) were diluted with BHI broth from this stock solution. *P. gingivalis* was grown in BHI broth with different concentrations of MSZ to report the growth of the bacteria (Bao et al., 2023). The control group was grown in BHI broth without MSZ. The blank well, which contained only BHI medium without inoculation of bacteria, was incubated under the same culture conditions as the experimental group. OD values at 600 nm were recorded from 0 to 72 h using Synergy HT (Agilent Technologies, Beijing, China) to describe the growth curves. To eliminate the influence of the culture medium on the absorbance value reading, the blank wells were used as the reference for zeroing. The minimum inhibitory concentration (MIC) values were the lowest concentrations of MSZ at which no visible growth was observed in the 15 mL sterile centrifuge tubes (BKMAM Biotechnology, Changshu, China) after incubation for 48 h.

### 2.3 Growth inhibition of MSZ against *Porphyromonas gingivalis* was assessed using flow cytometry

The percentage of live cells was assessed by flow cytometry assay (He et al., 2017). After 48 h of MSZ treatment, the *P. gingivalis* suspensions were added to 1 mL tubes and stained with SYTO (KeyGEN Biotech, Jiangsu, China) and PI (Beyotime, Shanghai, China) under light avoidance conditions for 15 min. Live/dead staining was used to differentiate between live and dead cells according to the kit protocols. A 0.9% sodium chloride solution was used as the flow cytometer sheath solution. Results were collected for 20,000 hits using FL1 through FL3 channels for each measurement point using the Accuri C6 Plus flow cytometer (BD Biosciences, USA). The results were assessed using FlowJo software (Tree Star, X.10.0.7, United States).

### 2.4 Effects of MSZ on the bacterial structure of *Porphyromonas gingivalis*

A total of 48 h after treatment, *P. gingivalis* suspensions were fixed in 2.5% glutaraldehyde and underwent dehydration through a graded ethanol series from 30 to 100%. Finally, the samples were gold-coated and observed using scanning electron microscopy (SEM) MAGNA (TeScan, Brno, Czech Republic). Atomic force microscopy (AFM) JPK Nano Wizard 3 (JPK Instruments, Berlin, Germany) in combination with an inverted Olympus IX81 light microscope, was used to examine the bacterial surface morphology. Roughness and Young’s modulus were analyzed using JPK Data Processing 7.0.97. *P. gingivalis* suspensions were placed on poly-L-lysine-coated (Sigma-Aldrich, Shanghai, China) culture plates for 20 min to fix (Benn et al., 2019). Cells were imaged in qualitative imaging mode using MLCT-A cantilevers (Bruker, MA, USA) with a spring constant of 0.07 N/m. The resonant frequency of the cantilever was 22 kHz in liquid.

### 2.5 *Porphyromonas gingivalis* biofilm formation assay and disruption of pre-formed *P. gingivalis* biofilms

The effect of MSZ on *P. gingivalis* biofilm formation was assessed by 3-(4,5-dimethylthiazol-2-yl)-2,5-diphenyl tetrazolium bromide (MTT) and crystal violet staining. Disruption of pre-formed *P. gingivalis* biofilms was also assessed by crystal violet staining. *P. gingivalis* biofilms were fixed with 2.5% glutaraldehyde and stained with 0.1% crystal violet reagent for 20 min, followed by 95% ethanol. OD values at 550 nm were measured to assess the effect of MSZ on *P. gingivalis* biofilm biomass. To evaluate *P. gingivalis* biofilm’s activity, 50 μL 0.5% MTT was added to *P. gingivalis* biofilms, anaerobically incubated in a dark place for 4 h, and then 100 μL of DMSO was added to dissolve the formazan crystals. The OD values at 490 nm were measured.

### 2.6 Anti-biofilm ability of MSZ

Confocal laser scanning microscopy (CLSM) was used to observe the effect of MSZ on both plaque biofilm formation and the disruption of established plaque biofilm. *P. gingivalis*, *F. nucleatum*, and *S. sanguinis* at a density of 1.0 × 10^8^ CFU/mL were inoculated to form a multispecies biofilm (Blanc et al., 2014). *F. nucleatum*, as an important bridging bacterium, can co-aggregate with early colonizing bacteria such as *S. sanguinis*, along with late colonizing bacteria like *P. gingivalis,* to form the plaque biofilm (Brennan and Garrett, 2019). *P. gingivalis* biofilms and multispecies biofilms were stained with SYTO/PI for 15 min in a dark place following the manufacturer’s instructions. Images were observed (490/635 nm for PI, 480/500 nm for SYTO) using FV3000 CLSM (Olympus, Tokyo, Japan). The percentage of live cells was analyzed using ImageJ2 2.3.0 software.

### 2.7 Inhibition of *Porphyromonas gingivalis* virulence gene expression treated with MSZ

Quantitative real-time polymerase chain reaction (qRT-PCR) was performed to investigate the expression of the *P. gingivalis* virulence gene using Mx3000P (Agilent Technologies, CA, USA). *P. gingivalis* plaque biofilms were scraped using a cell scraper (Thermo Fisher Scientific, Shanghai, China) and suspended in sterile PBS. After collection, lysozyme (Solarbio, Beijing, China) and RNAiso Plus (Takara Biomedical Technology, Beijing, China) were added to extract RNA. The lysozyme (12650-88-3, Solarbio, Beijing, China) was used at a concentration of 0.5 mg/mL. Each sample was added with 1 mL of RNAiso Plus (Trizol). cDNA synthesis was performed with Hifair® III 1st Strand cDNA Synthesis SuperMix (Yeasen, Shanghai, China), and qRT–PCR was performed with Hieff® qPCR SYBR Green Master Mix (Yeasen, Shanghai, China) following the manufacturer’s protocol. The total reaction volume was 20 μL and was completed on ice. The reaction program was 10 s at 95°C and 30 s at 60°C for 40 cycles. The 16S rRNA gene was used as the housekeeping amplicon. The levels of mRNA expression were calculated using the 2-ΔΔCt method. Primers (He et al., 2022) were obtained from Bioengineering (Shanghai) Co., Ltd., with the sequences detailed in Table 1.

**Table 1:** Primer sequences for qRT-PCR of *Porphyromonas gingivalis* virulence factors.

Gene	Forward primer sequence (5′ to 3′)	Reverse primer sequence (5′ to 3′)
16S rRNA	TGTAGATGACTGATGGTGAAA	ACTGTTAGCAACTACCGATGT
hagB	TGTCGCACGGCAAATATCGCTAAAC	CTGGCTGTCCTCGTCGAAAGCATAC
kgp	AGGAACGACAAACGCCTCTA	GTCACCAACCAAAGCCAAGA
rgpA	CACCGAAGTTCAAACCCCTA	GAGGGTGCAATCAGGACATT
rgpB	GCTCGGTCAGGCTCTTTGTA	GGGTAAGCAGATTGGCGATT
vimA	TCGCGTAGTCTGAGAGTAACCTT	GGTATAAACGAAGACAGCACGAC
mfa1	ACTTCTCCCGATTCATGGTG	GGATTCGGGTCAGGGTTATT

### 2.8 Arg-gingipain activity of *Porphyromonas gingivalis* assessed by BAPNA assay

The chromogenic substrate N-Benzoyl-L-arginine p-nitroanilide (BAPNA) kit (Solarbio, Beijing) was used to assess Arg-gingipain activity (Zhou et al., 2016; Shenbakam et al., 2021). *P. gingivalis* suspensions (10 μL each) were cultured in a buffer containing 200 mM Tris–HCl, 100 mM NaCl, and 5 mM CaCl_2_ at pH 7 to an OD_660_ of 0.5. The activity of the pre-incubated samples was then determined using 0.5 mM chromogenic substrate. The OD values were measured at 409 nm. The degradation observed after a 1-h treatment without drugs was set as the 100% reference value.

### 2.9 Cell membrane and wall integrity of *Porphyromonas gingivalis*

Alkaline phosphatase (AKP) and lactate dehydrogenase (LDH) activities were measured to evaluate the impact of MSZ on cell walls and membrane integrity (Li et al., 2024). *P. gingivalis* cultures in the logarithmic growth phase were centrifuged at 8,500 r/min for 5 min. After centrifugation, the samples were washed and resuspended in PBS. The bacteria were disrupted in an ice bath using the ultrasonic homogenizer JY92-IIDN (Scientz Biotechnology, Ningbo, China), and then the supernatant was collected by centrifugation at 15,000 r/min. A bicinchoninic protein assay kit (NCM Biotech, Suzhou, China) was used to determine the concentration of proteins. The AKP and LDH activities in the supernatant were determined according to the kit scheme (Jiancheng Bioengineering Institute, Nanjing, China). The OD values at 520 nm were measured for AKP activity, and the OD values at 440 nm were measured for LDH activity.

### 2.10 Rat periodontitis model

A total of 18 8-week-old male SD rats were randomly assigned to three groups (for each group, *n* = 6), i.e., the control group, the model group, and the MSZ group (treated with 1 mg/mL MSZ). Periodontitis models were established in the model group and MSZ group. Rats in the control group were anesthetized but not operated to exclude the effect of anesthesia. Rats were anesthetized by isoflurane (RWD Life Science, Shenzhen, China) inhalation (5% for induction and 2% for maintenance) using an anesthesia machine (Yuyan Instruments, Shanghai). An oral health exam was performed before the establishment of the rat periodontitis model. After anesthesia, a steel orthodontic wire with a diameter of 0.2 mm was inserted into the gap between the first molar and second molar, and the ligature was completely inserted into the gingival groove (Xu et al., 2019). The rats were checked every 72 h, and if the wires became loose, they were promptly re-tied. The ligature wire was set for 6 weeks. The success of the periodontitis model was judged by the depth of periodontal probing. After successful modeling, the ligature wires were removed. The rats in the MSZ group were injected with 20 μL of 1 mg/mL MSZ into the submucoperiosteal tissues at the midpoints of the buccal and palatal aspects of the maxillary first molars with an insulin syringe three times weekly for 2 weeks. Rats in both the control and model groups were injected with equal amounts of normal saline.

### 2.11 Micro-CT analysis

After 2 weeks of treatment, the rats were euthanized with carbon dioxide. The maxillae were collected and fixed in a 4% paraformaldehyde solution. The samples were scanned using micro-CT (μCT50, Scanco Medical AG, Switzerland). Scanning parameters were 114 mA, 70 kVp, and exposure time 0.3 s. The layer thickness was 18 μm. The focal area was the alveolar bone around the maxillary first molar. Using Image J2 2.3.0 software, we measured the distance of alveolar bone loss from the cementoenamel junction (CEJ) to the alveolar bone crest (ABC) at three anatomical sites (proximal, middle, and distal) on the palatal side of the maxillary first molar. In addition, the alveolar bone between the maxillary first and second molars was measured. After that, we used μCT Ray V4.2 software parameters to assess the amount of bone and mineralization, including bone volume fraction (bone volume/tissue volume, BV/TV, %), trabecular thickness (Tb. Th, mm), and trabecular separation (Tb. Sp, mm).

### 2.12 Hematoxylin–eosin staining

Rat maxillary tissue blocks were fixed in 10% paraformaldehyde for 48 h and then decalcified in 10% EDTA for 3 months, with the solution changed three times weekly. After decalcification, the samples were washed under running water for 20 min, trimmed, and sectioned into appropriate sizes. They were dehydrated using graded ethanol solutions, embedded in paraffin wax and sliced uniformly along the long axis of the teeth on the proximal-medial and distal-medial sides at a thickness of approximately 5 mm. The sections were deparaffinized in xylene, sealed in neutral gum, and stained with hematoxylin–eosin (H&E).

### 2.13 16S rRNA sequencing

The gingival tissues were rapidly and aseptically isolated and then frozen in liquid nitrogen for transport. Community genomic DNA was extracted using the E.Z.N.A™ Mag-Bind Soil DNA Kit (Omega, M5635-02, United States) according to the manufacturer’s instructions. We focused on the V3–V4 hypervariable region of the bacterial 16S rRNA gene. The sequencing was conducted at Sangon Biotech (Shanghai) Co., Ltd.

### 2.14 Statistical analyses

Data analysis was conducted using GraphPad Prism 9.3.1, with the results presented as mean ± standard deviation (SD). Each experiment was conducted for a minimum of three times. A t-test was used for comparisons between two groups, while analysis of variance (ANOVA) was employed for comparisons between multiple groups. Significance was expressed as *p* < 0.05 for statistical differences.

## 3 Results

### 3.1 MSZ inhibited the growth of *Porphyromonas gingivalis*

**Figure 1:**
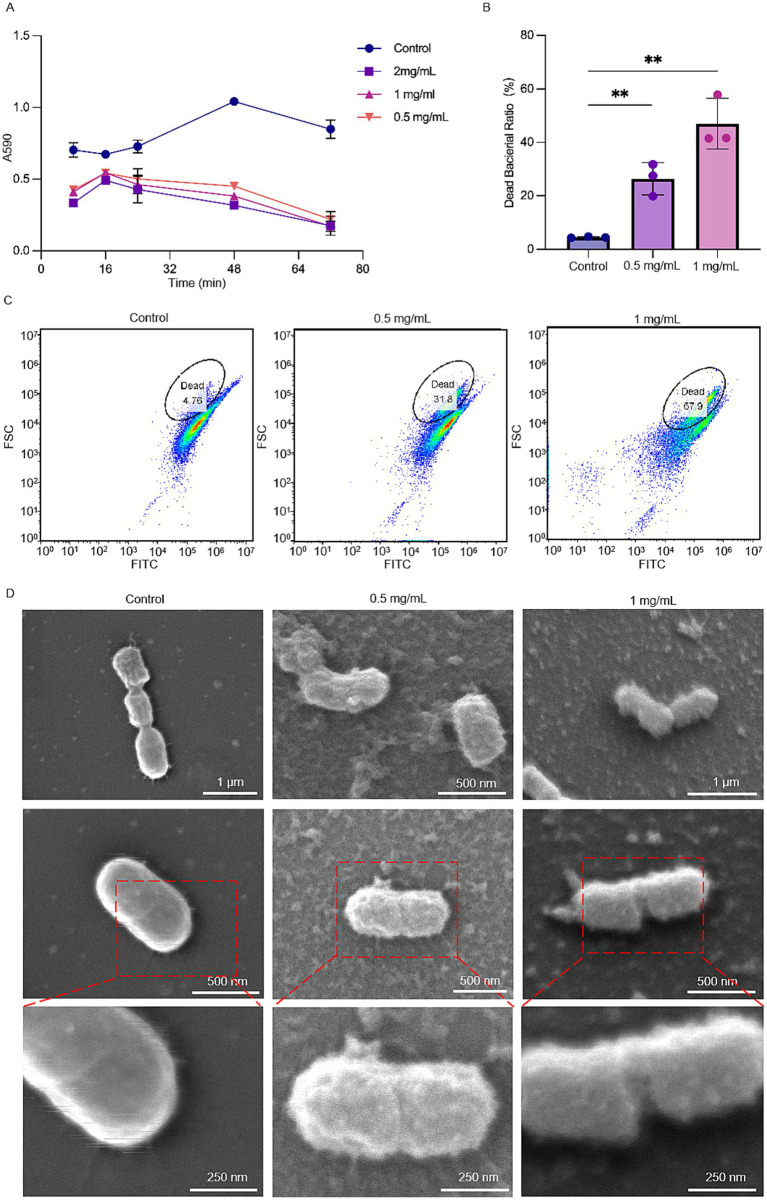
Effect of different concentrations of MSZ on the growth activity of *Porphyromonas gingivalis*. **(A)** Growth curve of *P. gingivalis* treated with different concentrations of MSZ. **(B)** Dead bacteria ratio for *P. gingivalis* in flow cytometry. **(C)** Flow cytometry of SYTO/PI-stained *P. gingivalis* with MSZ treatment. **(D)** SEM of *P. gingivalis* treated with different concentrations of MSZ. Bars marked with (**) show a significant difference at *p* < 0.01.

The growth curve showed that various concentrations of MSZ (0.5, 1, and 2 mg/mL) inhibited the growth of *P. gingivalis* compared to the control group, with a MIC value of 2 mg/mL (Figure 1A). To investigate MSZ’s effect on biofilm formation and to minimize the impact of its antibacterial effect, concentrations were kept below the MIC. Flow cytometry was used to assess *P. gingivalis* activity in both the experimental and control groups (Figures 1B,C). The percentage of dead cells significantly increased following treatment with MSZ 0.5 mg/mL and 1 mg/mL, compared to the control group.

Using SEM, the morphology of *P. gingivalis* cells was observed (Figure 1D). After 48 h of co-culture with MSZ at concentrations of 0.5 mg/mL and 1 mg/mL, *P. gingivalis* exhibited irreversible morphological changes, including invagination, wrinkling, breakage, atrophy, and deformation. In contrast, the untreated *P. gingivalis* cells maintained a rounded surface, intact structure, neat edges without wrinkles, and clearly visible bacterial pili. These observations suggest that MSZ concentrations of 0.5 mg/mL and 1 mg/mL may have damaged the surface structure and morphology of *P. gingivalis*. Treatment with 1 mg/mL MSZ transformed the initially smooth outer membrane into a broken and rough surface. The increased permeability of the cell membrane led to extracellular leakage of cellular contents, ultimately resulting in cell rupture and death. These findings indicate that MSZ may inhibit bacterial growth by compromising cell structure.

### 3.2 MSZ inhibited the formation of plaque biofilms and destructed pre-formed ones

**Figure 2:**
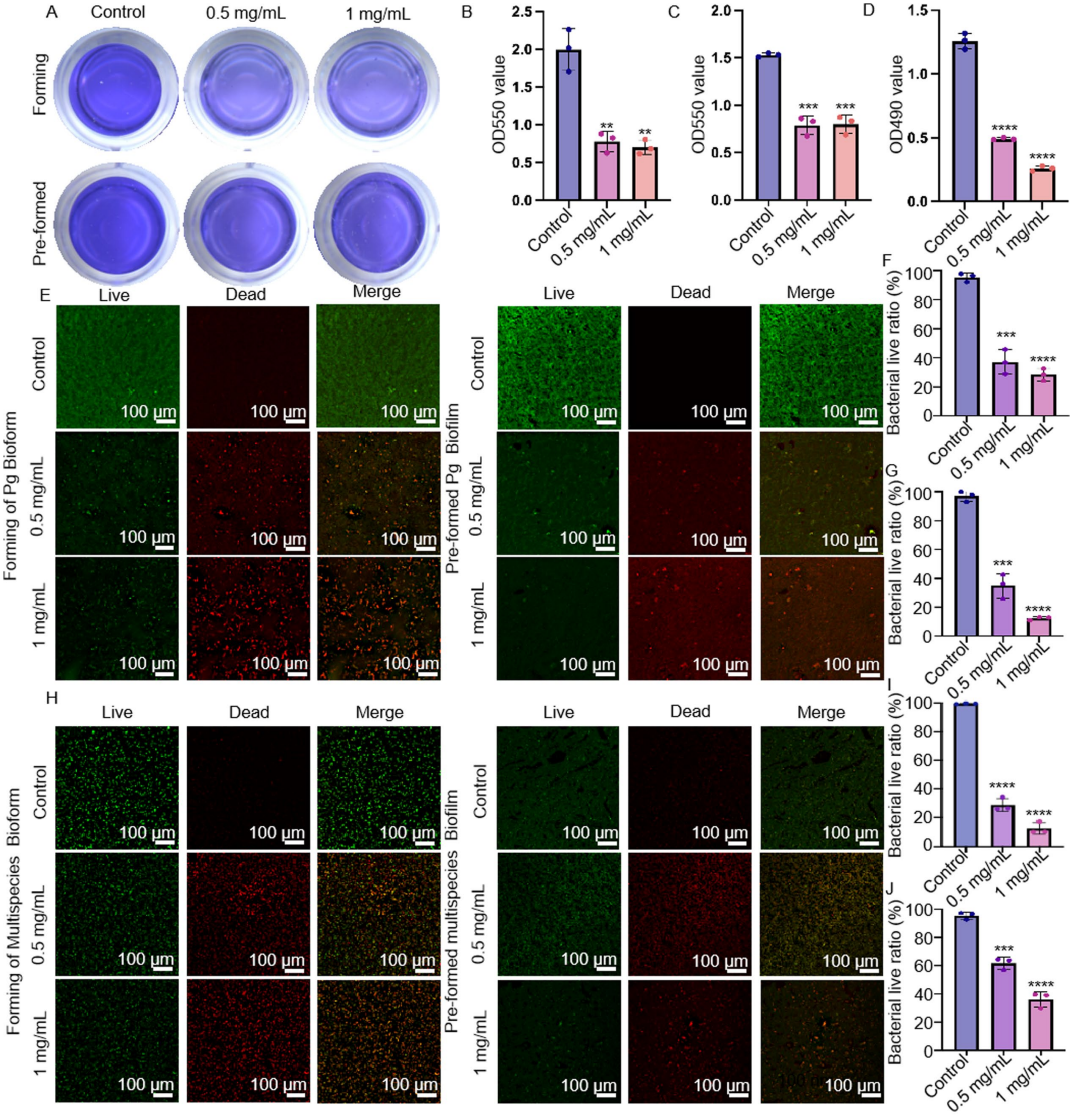
Preventive anti-biofilm potential of MSZ. **(A)** Effect of 0.5 mg/mL and 1 mg/mL MSZ on the overall biomass of *P. gingivalis* plaque biofilm formation and established plaque biofilms. **(B)** Histograms of the overall biomass of *P. gingivalis* plaque biofilm formation. **(C)** Histograms depicting total biomass distribution of pre-formed plaque biofilm spread. **(D)** Metabolic activity during plaque biofilm formation and **(E)** CLSM images of *P. gingivalis* plaque biofilm formation, including pre-formed plaque biofilms treated with varying MSZ concentrations. **(F)** Live bacteria ratio for *P. gingivalis* during plaque biofilm formation. **(G)** Live bacteria ratio for *P. gingivalis* in established plaque biofilm. **(H)** CLSM images depicting multispecies plaque biofilm formation and the effects of varying MSZ concentrations on pre-formed plaque biofilms. **(I)** Live bacteria ratio of multispecies plaque biofilm formation. **(J)** Live bacteria ratio of pre-formed multispecies plaque biofilm. Bars marked with (**), (***), and (****) represent significant differences at *p* < 0.01, *p* < 0.001, and *p* < 0.0001, respectively.

MSZ was added both before and after biofilm formation to simulate the prevention and treatment of biofilm. Crystal violet staining results showed that the OD_550 nm_ values decreased following treatment with 0.5 mg/mL and 1 mg/mL MSZ, indicating reduced biomass of *P. gingivalis* (Figures 2A–C). MTT analysis revealed that the activity of *P. gingivalis* biofilms was inhibited by MSZ at these concentrations (Figure 2D). The *P. gingivalis* and polymicrobial biofilms were also observed by CLSM (Figures 2E,H). In the control group, the biofilms were thick and densely structured. However, after 2 days of 0.5 mg/mL and 1 mg/mL MSZ treatment, the percentage of live cells was significantly decreased in both newly formed and established plaque biofilms (Figures 2F,G,I,J).

### 3.3 MSZ disrupted the bacterial structure of *Porphyromonas gingivalis* and inhibited the expression of *Porphyromonas gingivalis* virulence factors

**Figure 3:**
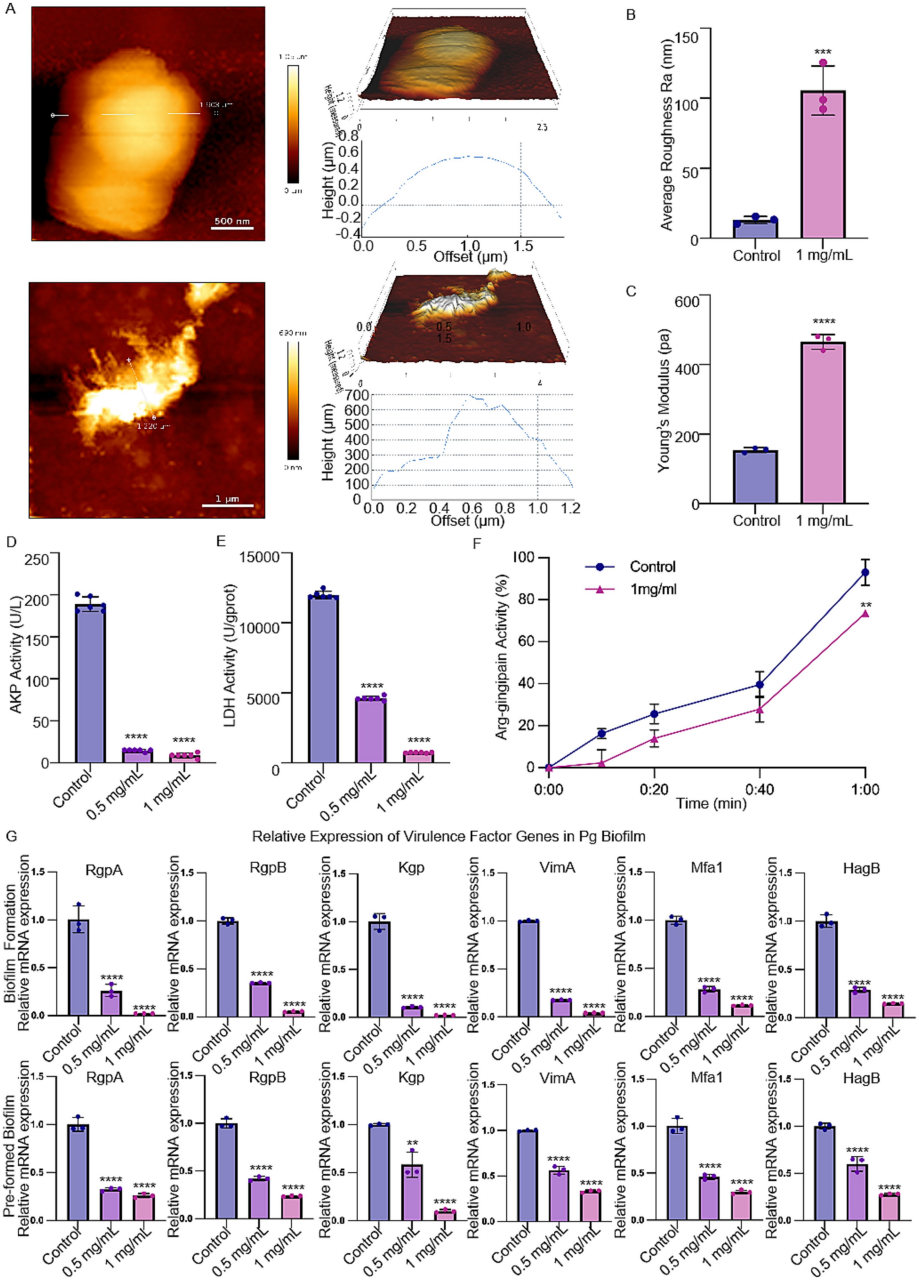
Effect of MSZ on *P. gingivalis* bacterial morphology structure and expression of virulence-associated genes. **(A)** AFM images of *P. gingivalis* and *P. gingivalis* treated by 1 mg/mL MSZ. **(B)** Change in *P. gingivalis* roughness affected by MSZ. **(C)** Change of Young’s modulus of *P. gingivalis* affected by MSZ. **(D)** Effect of MSZ on *P. gingivalis* Akp activity. **(E)** Effect of MSZ on *P. gingivalis* LDH activity. **(F)** Effect of MSZ on *P. gingivalis* gingipain activity. The degradation observed after a 1-h treatment without drugs was set as the 100% reference value. **(G)** Analysis of relative mRNA levels of virulence factor genes in *P. gingivalis* during plaque biofilm formation and in pre-formed plaque biofilm using RT-qPCR. Bars marked with (**), (***), and (****) represent significant differences at *p* < 0.01, *p* < 0.001, and *p* < 0.0001, respectively.

The structure of *P. gingivalis* was observed by AFM. After 48 h of treatment with 1 mg/mL MSZ, the diameter of *P. gingivalis* decreased, and the surface became rough and irregular (Figure 3A). The average roughness significantly increased compared to untreated cells (Figure 3B). Young’s modulus was increased after 48 h of treatment, indicating that MSZ reduced the elasticity of the cell membrane (Figure 3C). In this study, we also determined the integrity of the cell wall using AKP analysis and the integrity of the cell membrane by assessing the LDH activity. Treatment with 0.5 mg/mL and 1 mg/mL MSZ led to a significant reduction of intracellular AKP and LDH activity in *P. gingivalis* compared to the control group (Figures 3D,E).

The effect of MSZ on the expression of virulence genes was assessed by qRT-PCR. The selected genes include a hemagglutinin B (HagB), a lysine-specific cysteine proteinase (Kgp), an arginine-specific cysteine proteinase A (RgpA), an arginine-specific cysteine proteinase B (RgpB), a minor fimbrial antigen (Mfa1), and virulence-modulating gene A (VimA). Treatment with MSZ at concentrations of 0.5 mg/mL and 1 mg/mL significantly reduced the expression of these *P. gingivalis* virulence genes in both biofilm-forming and pre-formed plaque biofilms (Figure 3G). The activity of gingipains was further evaluated using a BAPNA assay, which showed that 1 mg/mL MSZ could decrease *P. gingivalis* gingipain activity (Figure 3F).

### 3.4 The therapeutic effect of MSZ on periodontitis

**Figure 4:**
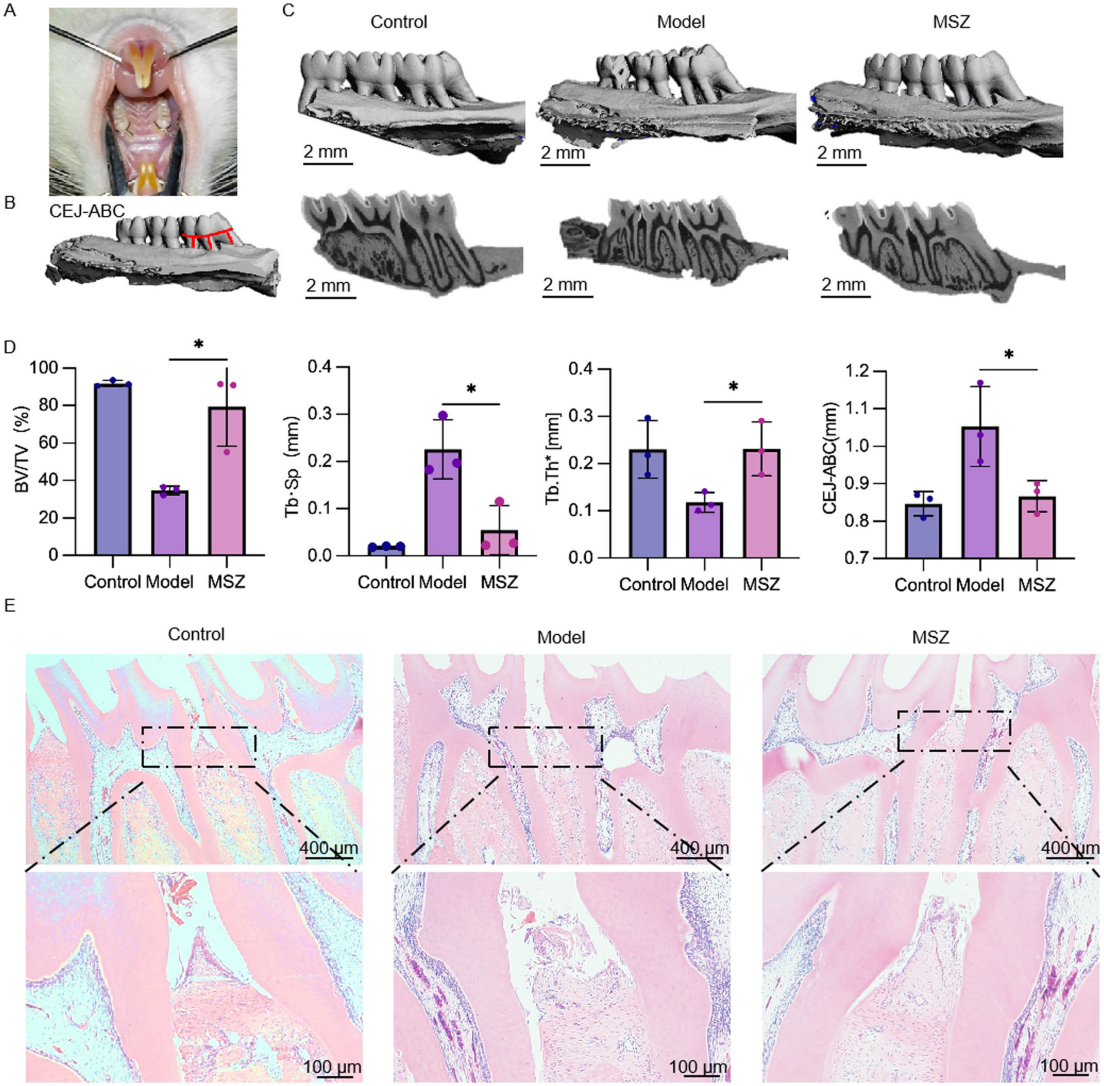
Effect of MSZ on the bone structure of the maxillary first molar in periodontitis rats. **(A)** Image of a periodontitis model. **(B)** Alveolar bone resorption of the maxillary first molar. The red line marks the CEJ-ABC distance. **(C)** Three-dimensional reconstruction and a sagittal micro-CT section of the maxillary first molar of rats in each group. **(D)** Micro-CT was used to analyze the bone structure parameters including BV/TV, Tb. Th, and Tb. Sp. The bar chart shows the CEJ-ABC distance. **(E)** H&E stained images of the periodontium were taken after 2 weeks post-treatment. First row of images (4×). Bars marked with (*) show a significant difference at *p* < 0.05.

Subsequently, we assessed the potential of MSZ to induce a comparable effect *in vivo*, which is crucial for periodontitis treatment. We developed a rat model to study experimental periodontitis (Figure 4A). Given the challenge of rapidly achieving an effective dose at the periodontal infection site with systemic medication, this study utilized local injections of MSZ. After euthanasia, maxillary samples from the rats were collected and analyzed using micro-CT scanning. Bone loss was assessed in three-dimensional palatal images by measuring the CEJ-ABC distance of the first molar’s distal buccal root (Figure 4B). Alveolar bone resorption was observed between the first and second maxillary molars following the installation of ligature wires. The model group showed increased bone loss on the palatal side compared to the controls (Figure 4C), confirming successful model establishment via the elevated CEJ-ABC distance. In the MSZ-treated group, the CEJ-ABC distance was significantly reduced compared to the model group, indicating less alveolar bone resorption. The model group exhibited decreased bone volume fraction and trabecular thickness and increased trabecular separation compared to the control group, confirming successful periodontitis modeling. In contrast, the MSZ group showed a significantly higher bone volume fraction and trabecular thickness, along with significantly lower trabecular separation, compared to the model group. In the model group, MSZ contributed to the partial restoration of bone volume (Figure 4D). Bone volume increased primarily due to reduced trabecular spacing and enhanced trabecular thickness.

To validate the results, the periodontal tissues from each rat group were assessed using H&E staining (Figure 4E). The H&E staining results indicated that the gingival papillae in the control group were tightly integrated with the epithelium and enamel surfaces. In contrast, the model group exhibited eroded gingival papillae, accompanied by epithelial proliferation toward the root and evident alveolar bone resorption, confirming the successful establishment of the periodontitis model. Compared to the model group, the MSZ-treated group showed reduced alveolar bone destruction and less damage to periodontal tissues, indicating that MSZ effectively controlled periodontitis in rats.

### 3.5 MSZ modulated periodontal flora composition in rats with periodontitis

**Figure 5:**
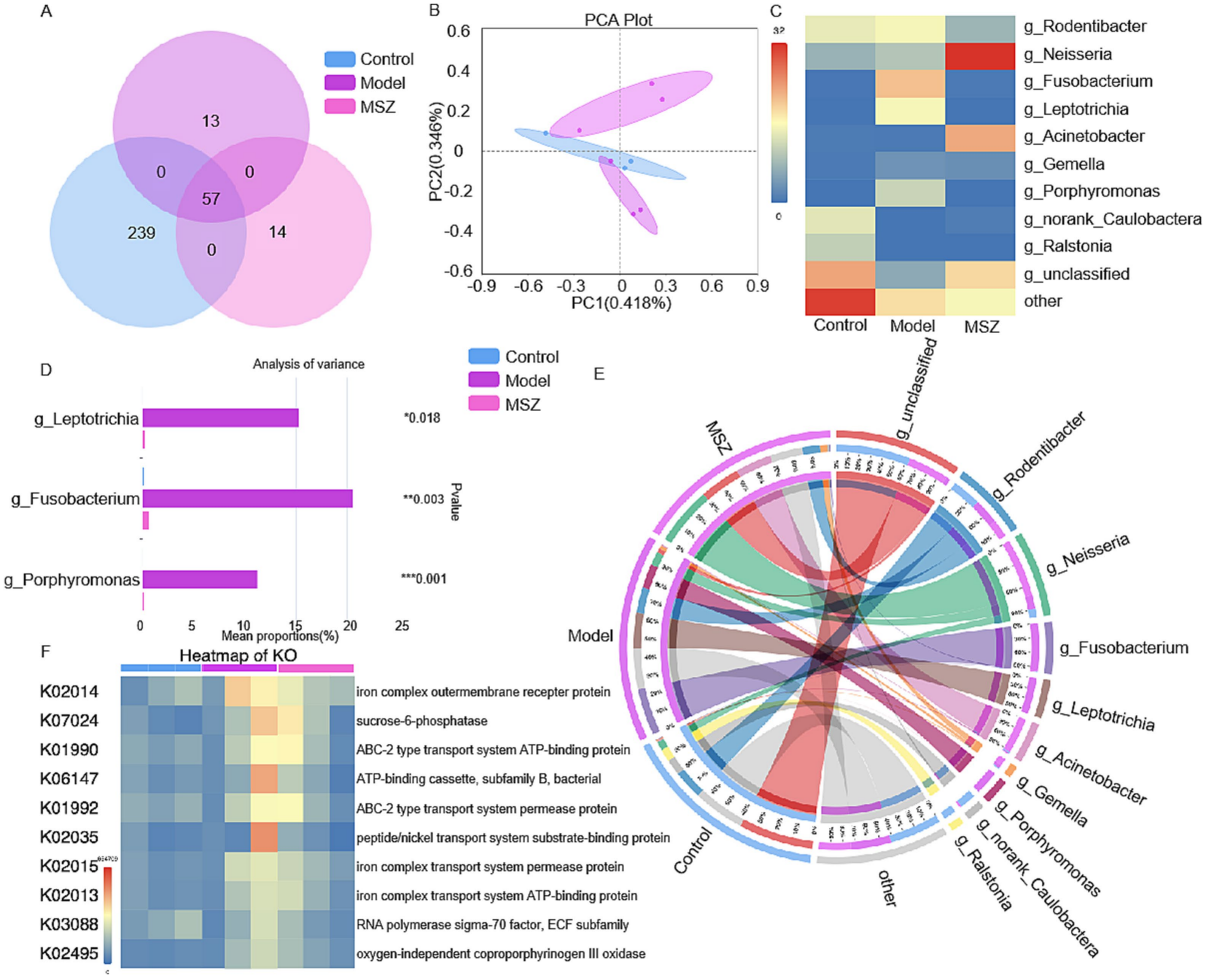
16S RNA sequencing of periodontal tissue flora in rats with periodontitis. **(A)** Venn diagram for the strains of identified bacteria in the three groups. **(B)** Principal component analysis plot of the differences between the three groups. All experiments were performed in three biological replicates. **(C)** Relative abundance of periodontal microbiota after MSZ treatment. **(D)** The proportion of three kinds of bacteria with significant differences in the periodontal microbiota after MSZ treatment. **(E)** Differences in periodontal microbiota of control, model, and MSZ groups. **(F)** KEGG functional prediction analysis. Bars marked with (*), (**), and (***) represent significant differences at *p* < 0.05, *p* < 0.01, and *p* < 0.001, respectively.

We examined the impact of MSZ treatment on the periodontal flora composition, which changes in the presence of periodontitis. *P. gingivalis* and *F. nucleatum* are the primary pathogens responsible for this condition. Using a ligature-induced rat model of periodontitis, we identified microorganisms from gingival tissues after 2 weeks of MSZ treatment by 16S rRNA sequencing. Our results showed that the control, model, and MSZ groups shared 57 common genera, with unique species totaling 239, 13, and 14 genera, respectively (Figure 5A). Principal component analysis indicated the differences in the species composition of the samples among the three groups (Figure 5B). In total, 10 bacterial species with significant differences and their proportion in the periodontal microbiota after MSZ treatment were showed in Figures 5C,E. The 16S rRNA identification results showed that the proportion of *Porphyromonas*, *Fusobacterium*, and *Leptotrichia* decreased after MSZ treatment when compared to the model group (Figure 5D). KEGG functional prediction analysis revealed that the most abundant KEGG categories were associated with the iron complex transport system, ABC-2 type transport system, sucrose-6-phosphatase, ATP-binding cassette, peptide/nickel transport system, RNA polymerase, and coproporphyrinogen oxidase (Figure 5F). In the complex oral environment, MSZ can reduce periodontal pathogens and disrupt plaque biofilm formation, leading to decreased local inflammation and prevention of further alveolar bone resorption.

## 4 Discussion

It has been reported that MSZ exhibits antibacterial activity, leading to the downregulation of genes involved in bacterial invasion (Kaufman et al., 2009). However, the underlying mechanism remains unclear. This study has shown that MSZ, as a non-steroidal anti-inflammatory drug, commonly used as a first-line treatment for IBD, is also effective in a rat periodontitis model and could help maintain the microbiota balance. Specifically, our findings showed that MSZ inhibited the formation of *P. gingivalis* biofilms and dispersed pre-formed plaque biofilms by impacting both the structural integrity of the plaque biofilm and the expression of biofilm-related genes. Notably, our results have shown that MSZ is a potential therapeutic agent for periodontitis.

To investigate the role of MSZ on bacterial growth, initial assays revealed complete suppression of visible growth at a concentration of 2 mg/mL (Figure 1A). The proportion of live cells to total cells following MSZ treatment decreased compared to untreated controls (Figures 1B,C). Previous studies have reported that MSZ has antibacterial activity against various pathogens, including *S. aureus*, *E. coli*, *P. aeruginosa*, and *B. subtilis*, and antifungal activity against *Candida albicans* and *Aspergillus fumigatus in vitro* (Soliman and Mohamed, 2013).

To date, the impact of topical periodontal application of MSZ on the periodontal microflora has not been studied. Using SEM and AFM, we observed that MSZ induced significant morphological changes in *P. gingivalis* cells. At a concentration of 0.5 mg/mL, MSZ began to degrade the cell wall, while at 1 mg/mL, it compromised cell membrane integrity (Figures 1D, 3A–C). The leakage of AKP and LDH from the intracellular space indicated a disruption of the bacterial cell barrier (Lang et al., 2023), as validated by AKP and LDH assays (Figures 3D,E). Collectively, our results demonstrated that *in situ* use of MSZ could effectively disrupt the bacterial cell wall and membrane, enhance permeability, and cause the release of intracellular contents.

Resistance to conventional antibiotics may increase due to the microbiota’s ability to form plaque biofilms (Noiri et al., 2003). This complex process, regulated by multiple signaling pathways, involves initial attachment, bacterial co-aggregation, maturation into a three-dimensional structure, and eventual detachment and recolonization (Li et al., 2022). In this study, MTT and crystal violet assays showed that MSZ disrupted both the formation of plaque biofilms and the destruction of pre-formed plaque biofilms (Figures 2A–D). The *P. gingivalis* and multispecies biofilms were also observed by CLSM (Figures 2E,H). The proportion of dead cells to total cells following MSZ treatment increased compared to untreated controls (Figures 2F,G,I,J).

To uncover the potential underlying pathway, qRT-PCR was examined, and the results showed that MSZ inhibited plaque biofilm formation, possibly by downregulating plaque biofilm-related genes (Figure 3G). Specifically, the VimA (Aruni et al., 2012; Aruni et al., 2013), Mfa1 (Nagano et al., 2013; How et al., 2016b; Lee et al., 2018) and HagB (Azelmat et al., 2015) genes, which play a vital role in mediating attachment, were downregulated by MSZ, thereby reducing bacterial adhesion and loosening the plaque biofilm structure. Gingipains, key virulence factors of *P. gingivalis*, are classified into Rgp and Kgp proteases, which are essential for evading the host defense system (Imamura, 2003; Olsen and Potempa, 2014). Our results showed that MSZ downregulated gingipain genes and inhibited the Rgp activity, thereby reducing the bacterial pathogenicity (Figure 3F). These results are consistent with previous studies demonstrating that MSZ can modify bacterial gene expression (Kaufman et al., 2009), inhibit bacterial folate biosynthesis (London, 2024), and suppress polyphosphate accumulation in bacteria (Dahl et al., 2017), thus altering bacterial colonization and plaque biofilm-forming capability. Moreover, a derivative of MSZ has been shown to suppress bacterial virulence (adherence, invasion, and translocation) through its iron-scavenging property (Motta et al., 2018). In summary, our results show that MSZ reduced the expression of plaque biofilm-related genes, ultimately loosening the plaque biofilm structure and decreasing bacterial pathogenicity, resulting in a reduction in the number of bacteria adhering to and invading the periodontal tissue. MSZ not only disrupts the biofilm but also penetrates its interior, ultimately leading to the killing of pathogenic bacteria.

In the *in vivo* experiment, we established a periodontitis model in rats using ligation. Micro-CT and HE staining revealed that MSZ significantly inhibited alveolar bone resorption and alleviated periodontal tissue inflammation (Figure 4). This therapeutic effect likely results from dual mechanisms: anti-inflammatory action and regulation of the microbiota. MSZ could inhibit pro-inflammatory mediators such as leukotrienes, prostaglandin, IL-1, NF-κB, and TNF-*α*, along with a PPAR-*γ* receptor agonist (Wada, 2023). Previous studies in our group have shown that MSZ inhibits pro-inflammatory factors and peroxides, demonstrating therapeutic efficacy in periodontitis. In this study, MSZ was found to improve the composition of the periodontal microbiota, promoting a healthier microbiota state as determined by 16S rRNA sequencing (Figure 5).

Previous research has focused on the effects of MSZ in restoring gut microbiota balance in IBD treatment. Studies have shown that, as a PPAR-γ agonist, MSZ can restore mitochondrial bioenergetics in the colonic epithelium by stimulating mitochondrial activity (Lee et al., 2020; Mei et al., 2022), thereby promoting beneficial bacteria and reducing harmful ones to regulate the intestinal microbiota (Wada, 2023). MSZ treatment partially restored the gut microbiota and fungal balance, reduced the abundance of pathogenic bacteria (such as *Bacillus*, *Proteobacteria,* and *P. gingivalis*), and increased the abundance of beneficial bacteria such as *Butyricicoccus, Parabacteroides,* and *Faecalibacterium*, as measured by 16S rRNA sequencing (Xue et al., 2013; Olaisen et al., 2019; El-Baz et al., 2020; Dai et al., 2021). In contrast, our study found that MSZ reduces the number of periodontal pathogens (such as *F. nucleatum* and *P. gingivalis*) and restores the balance of periodontal microbiota, providing evidence for the treatment of IBD and periodontitis (Figure 5D). According to the KEGG pathway analysis, MSZ were found to target vital proteins involved in membrane transport of iron, carbohydrate metabolism, and virulence of periodontal pathogens (Figure 5F). The acquisition of iron and hemin is crucial for the metabolism and virulence of *P. gingivalis* (Anaya-Bergman et al., 2015; Smalley and Olczak, 2017). Our results are consistent with the findings of previous *in vitro* experiments (Figure 3G).

MSZ exhibits a variety of pharmacologic activities, and this study demonstrated its anti-biofilm properties, providing a novel therapeutic option for periodontitis. However, the current experiment has certain limitations. The rat periodontitis model has demonstrated the therapeutic potential of MSZ for periodontitis. This study was limited to *in vitro* and *in vivo* animal models. Clinical studies are an important aspect that requires further exploration. The molecular targets for MSZ needed further research. Notably, the topical administration of MSZ for periodontitis treatment requires further exploration and refinement. Our group plans to develop a more stable and effective local delivery system in future experiments. Additionally, future research could focus on optimizing the molecular structure of MSZ to identify the most efficacious compound for clinical trials and thoroughly evaluate its therapeutic potential.

In conclusion, our study has demonstrated that MSZ could loosen the structure of oral plaque biofilms, downregulate the expression of biofilm-related genes, regulate the periodontal microecological balance, hinder the formation and accumulation of dental plaque, reduce plaque and pathogenic bacteria, alleviate local inflammation, inhibit alveolar bone resorption, and thus control the development of periodontitis. This study not only expands the application range of MSZ but also provides a new idea for the treatment of periodontitis. Meanwhile, this study demonstrates for the first time the therapeutic potential of MSZ for periodontitis, thus adding new therapeutic evidence to the relationship between periodontitis and IBD. In spite of this, the antibacterial mechanism and efficacy of MSZ in the treatment of periodontitis still require in-depth investigation and verification to clarify its clinical value.

## Data Availability

The original contributions presented in the study are included in the article/supplementary material, further inquiries can be directed to the corresponding authors.

## References

[ref1] Anaya-Bergman C. Rosato A. Lewis J. P. (2015). Iron-and hemin-dependent gene expression of *Porphyromonas gingivalis*. Mol Oral Microbiol 30, 39–61. doi: 10.1111/omi.12066, PMID: 25043610

[ref2] Aruni A. W. Lee J. Osbourne D. Dou Y. Roy F. Muthiah A.. (2012). VimA-dependent modulation of acetyl coenzyme a levels and lipid a biosynthesis can Alter virulence in *Porphyromonas gingivalis*. Infect. Immun. 80, 550–564. doi: 10.1128/IAI.06062-11, PMID: 22144476 PMC3264320

[ref3] Aruni A. W. Robles A. Fletcher H. M. (2013). VimA mediates multiple functions that control virulence in *Porphyromonas gingivalis*. Mol Oral Microbiol 28, 167–180. doi: 10.1111/omi.12017, PMID: 23279905 PMC3625487

[ref4] Azelmat J. Larente J. F. Grenier D. (2015). The anthraquinone rhein exhibits synergistic antibacterial activity in association with metronidazole or natural compounds and attenuates virulence gene expression in *Porphyromonas gingivalis*. Arch. Oral Biol. 60, 342–346. doi: 10.1016/j.archoralbio.2014.11.006, PMID: 25463909

[ref5] Bao S. Sun S. Li L. Xu L. (2023). Synthesis and antibacterial activities of ag-TiO(2)/ZIF-8. Front. Bioeng. Biotechnol. 11:1221458. doi: 10.3389/fbioe.2023.1221458, PMID: 37576996 PMC10415108

[ref6] Benn G. Pyne A. L. B. Ryadnov M. G. Hoogenboom B. W. (2019). Imaging live bacteria at the nanoscale: comparison of immobilisation strategies. Analyst 144, 6944–6952. doi: 10.1039/C9AN01185D, PMID: 31620716 PMC7138128

[ref7] Blanc V. Isabal S. Sánchez M. C. Llama-Palacios A. Herrera D. Sanz M.. (2014). Characterization and application of a flow system for in vitro multispecies oral biofilm formation. J. Periodontal Res. 49, 323–332. doi: 10.1111/jre.12110, PMID: 23815431

[ref8] Brennan C. A. Garrett W. S. (2019). *Fusobacterium nucleatum* — symbiont, opportunist and oncobacterium. Nat. Rev. Microbiol. 17, 156–166. doi: 10.1038/s41579-018-0129-6, PMID: 30546113 PMC6589823

[ref9] Cevallos S. A. Lee J.-Y. Velazquez E. M. Foegeding N. J. Shelton C. D. Tiffany C. R.. (2021). 5-Aminosalicylic acid ameliorates colitis and checks Dysbiotic *Escherichia coli* expansion by activating PPAR-γ signaling in the intestinal epithelium. MBio 12, e03227–e03220. doi: 10.1128/mBio.03227-20 33468700 PMC7845635

[ref10] Dahl J.-U. Gray M. J. Bazopoulou D. Beaufay F. Lempart J. Koenigsknecht M. J.. (2017). The anti-inflammatory drug mesalamine targets bacterial polyphosphate accumulation. Nat. Microbiol. 2:16267. doi: 10.1038/nmicrobiol.2016.267 28112760 PMC5514548

[ref11] Dai L. Tang Y. Zhou W. Dang Y. Sun Q. Tang Z.. (2021). Gut microbiota and related metabolites were disturbed in ulcerative colitis and partly restored after Mesalamine treatment. Front. Pharmacol. 11:620724. doi: 10.3389/fphar.2020.620724, PMID: 33628183 PMC7898679

[ref12] De Souza H. S. P. Fiocchi C. (2016). Immunopathogenesis of IBD: current state of the art. Nat. Rev. Gastroenterol. Hepatol. 13, 13–27. doi: 10.1038/nrgastro.2015.186, PMID: 26627550

[ref13] El-Baz A. M. Khodir A. E. Adel El-Sokkary M. M. Shata A. (2020). The protective effect of Lactobacillus versus 5-aminosalicylic acid in ulcerative colitis model by modulation of gut microbiota and Nrf2/ho-1 pathway. Life Sci. 256:117927. doi: 10.1016/j.lfs.2020.117927, PMID: 32526285

[ref14] Gatej S. M. Marino V. Bright R. Fitzsimmons T. R. Gully N. Zilm P.. (2018). Probiotic *Lactobacillus rhamnosus* GG prevents alveolar bone loss in a mouse model of experimental periodontitis. J. Clin. Periodontol. 45, 204–212. doi: 10.1111/jcpe.12838, PMID: 29121411

[ref15] He S. Hong X. Huang T. Zhang W. Zhou Y. Wu L.. (2017). Rapid quantification of live/dead lactic acid bacteria in probiotic products using high-sensitivity flow cytometry. Methods Appl. Fluoresc. 5:024002. doi: 10.1088/2050-6120/aa64e4, PMID: 28357994

[ref16] He Z. Jiang W. Jiang Y. Dong J. Song Z. Xu J.. (2022). Anti-biofilm activities of coumarin as quorum sensing inhibitor for *Porphyromonas gingivalis*. J. Oral Microbiol. 14:2055523. doi: 10.1080/20002297.2022.2055523, PMID: 35368854 PMC8967191

[ref17] How K. Y. Song K. P. Chan K. G. (2016b). Porphyromonas gingivalis: An Overview of Periodontopathic Pathogen below the Gum Line. Front Microbiol 7:53. doi: 10.3389/fmicb.2016.00053, PMID: 26903954 PMC4746253

[ref18] Huang L. Zheng J. Sun G. Yang H. Sun X. Yao X.. (2022). 5-Aminosalicylic acid ameliorates dextran sulfate sodium-induced colitis in mice by modulating gut microbiota and bile acid metabolism. Cell. Mol. Life Sci. 79:460. doi: 10.1007/s00018-022-04471-3, PMID: 35913641 PMC11071811

[ref19] Imai J. Ichikawa H. Kitamoto S. Golob J. L. Kaneko M. Nagata J.. (2021). A potential pathogenic association between periodontal disease and Crohn’s disease. JCI Insight 6:e148543. doi: 10.1172/jci.insight.148543, PMID: 34710061 PMC8675195

[ref20] Imamura T. (2003). The role of gingipains in the pathogenesis of periodontal disease. J. Periodontol. 74, 111–118. doi: 10.1902/jop.2003.74.1.111, PMID: 12593605

[ref21] Jia L. Jiang Y. Wu L. Fu J. Du J. Luo Z.. (2024). *Porphyromonas gingivalis* aggravates colitis via a gut microbiota-linoleic acid metabolism-Th17/Treg cell balance axis. Nat. Commun. 15:1617. doi: 10.1038/s41467-024-45473-y, PMID: 38388542 PMC10883948

[ref22] Kang E. A. Chun J. Kim J. H. Han K. Soh H. Park S.. (2020). Periodontitis combined with smoking increases risk of the ulcerative colitis: a national cohort study. WJG 26, 5661–5672. doi: 10.3748/wjg.v26.i37.5661, PMID: 33088159 PMC7545388

[ref23] Kaufman J. Griffiths T. A. Surette M. G. Ness S. Rioux K. P. (2009). Effects of mesalamine (5-aminosalicylic acid) on bacterial gene expression. Inflamm. Bowel Dis. 15, 985–996. doi: 10.1002/ibd.20876, PMID: 19202572

[ref24] Lang A. Lan W. Xie J. (2023). Preparation and antimicrobial mechanism of Maillard reaction products derived from ε-polylysine and chitooligosaccharides. Biochem. Biophys. Res. Commun. 650, 30–38. doi: 10.1016/j.bbrc.2023.01.078, PMID: 36773337

[ref25] Lee J.-Y. Cevallos S. A. Byndloss M. X. Tiffany C. R. Olsan E. E. Butler B. P.. (2020). High-fat diet and antibiotics cooperatively impair mitochondrial bioenergetics to trigger Dysbiosis that exacerbates pre-inflammatory bowel disease. Cell Host Microbe 28:e6. doi: 10.1016/j.chom.2020.06.001 PMC7429289 32668218

[ref26] Lee Y.-C. Liu C.-Y. Lee C.-L. Zhang R.-H. Huang C.-J. Yen T.-L. (2022). The Periodontopathic pathogen, *Porphyromonas gingivalis*, involves a gut inflammatory response and exacerbates inflammatory bowel disease. Pathogens 11:84. doi: 10.3390/pathogens11010084, PMID: 35056032 PMC8779656

[ref27] Lee J. Y. Miller D. P. Wu L. Casella C. R. Hasegawa Y. Lamont R. J. (2018). Maturation of the Mfa1 fimbriae in the Oral pathogen *Porphyromonas gingivalis*. Front. Cell. Infect. Microbiol. 8:137. doi: 10.3389/fcimb.2018.00137, PMID: 29868494 PMC5954841

[ref28] Li Z. He Q. Xu F. Yin X. Guan Z. Song J.. (2024). Exploring the antibacterial potential and underlying mechanisms of *Prunella vulgaris* L. on methicillin-resistant *Staphylococcus aureus*. Food Secur. 13:660. doi: 10.3390/foods13050660, PMID: 38472772 PMC10931123

[ref29] Li X. Liu Y. Yang X. Li C. Song Z. (2022). The Oral microbiota: community composition, influencing factors, pathogenesis, and interventions. Front. Microbiol. 13:895537. doi: 10.3389/fmicb.2022.895537, PMID: 35572634 PMC9100676

[ref30] Lira-Junior R. Figueredo C. M. (2016). Periodontal and inflammatory bowel diseases: is there evidence of complex pathogenic interactions? World J. Gastroenterol. 22, 7963–7972. doi: 10.3748/wjg.v22.i35.7963, PMID: 27672291 PMC5028810

[ref31] Liu H. Xu Y. Cui Q. Liu N. Chu F. Cong B.. (2021). Effect of psoralen on the intestinal barrier and alveolar bone loss in rats with chronic periodontitis. Inflammation 44, 1843–1855. doi: 10.1007/s10753-021-01462-7, PMID: 33839980

[ref32] London R. E. (2024). The aminosalicylate - folate connection. Drug Metab. Rev. 56, 80–96. doi: 10.1080/03602532.2024.2303507, PMID: 38230664 PMC11305456

[ref33] Madsen G. R. Bertl K. Pandis N. Stavropoulos A. Burisch J. (2023). The impact of periodontitis on inflammatory bowel disease activity. Inflamm. Bowel Dis. 29, 396–404. doi: 10.1093/ibd/izac090, PMID: 35552410

[ref34] Mei X. Mell B. Manandhar I. Aryal S. Tummala R. Kyoung J.. (2022). Repurposing a drug targeting inflammatory bowel disease for lowering hypertension. JAHA 11:e027893. doi: 10.1161/JAHA.122.027893, PMID: 36533597 PMC9798790

[ref35] Mishra S. (2021). Role of probiotics in adjunct to non-surgical periodontal therapy in patients with chronic periodontitis: a systematic review and meta-analysis. J. Biol. Regul. Homeost. Agents 35, 67–78. doi: 10.23812/21-2supp1-6, PMID: 34281303

[ref36] Motta J.-P. Allain T. Green-Harrison L. E. Groves R. A. Feener T. Ramay H.. (2018). Iron sequestration in microbiota biofilms as a novel strategy for treating inflammatory bowel disease. Inflamm. Bowel Dis. 24, 1493–1502. doi: 10.1093/ibd/izy116, PMID: 29788224 PMC5995063

[ref37] Nagano K. Abiko Y. Yoshida Y. Yoshimura F. (2013). Genetic and antigenic analyses of *Porphyromonas gingivalis* Fim a fimbriae. Mol Oral Microbiol 28, 392–403. doi: 10.1111/omi.12032, PMID: 23809984

[ref38] Noiri Y. Okami Y. Narimatsu M. Takahashi Y. Kawahara T. Ebisu S. (2003). Effects of chlorhexidine, minocycline, and metronidazole on *Porphyromonas gingivalis* strain 381 in biofilms. J. Periodontol. 74, 1647–1651. doi: 10.1902/jop.2003.74.11.1647, PMID: 14682662

[ref39] Olaisen M. Spigset O. Flatberg A. Granlund A. V. B. Brede W. R. Albrektsen G.. (2019). Mucosal 5-aminosalicylic acid concentration, drug formulation and mucosal microbiome in patients with quiescent ulcerative colitis. Aliment. Pharmacol. Ther. 49, 1301–1313. doi: 10.1111/apt.15227, PMID: 30895635 PMC6593792

[ref40] Olsen I. Potempa J. (2014). Strategies for the inhibition of gingipains for the potential treatment of periodontitis and associated systemic diseases. J. Oral Microbiol. 6:24800. doi: 10.3402/jom.v6.24800, PMID: 25206939 PMC4138498

[ref41] Shaw K. A. Bertha M. Hofmekler T. Chopra P. Vatanen T. Srivatsa A.. (2016). Dysbiosis, inflammation, and response to treatment: a longitudinal study of pediatric subjects with newly diagnosed inflammatory bowel disease. Genome Med. 8:75. doi: 10.1186/s13073-016-0331-y, PMID: 27412252 PMC4944441

[ref42] Shenbakam P. Rao R. J. Prabhu S. Srirangarajan S. Rudresh V. (2021). Influence of antibacterial effects of tetracycline, laser, and photodynamic therapy on cell viability, cell damage, and virulence of *Porphyromonas gingivalis*. Photodiagn. Photodyn. Ther. 36:102617. doi: 10.1016/j.pdpdt.2021.102617, PMID: 34740837

[ref43] Slots J. (2017). Periodontitis: facts, fallacies and the future. Periodontology 75, 7–23. doi: 10.1111/prd.12221, PMID: 28758294

[ref44] Smalley J. W. Olczak T. (2017). Heme acquisition mechanisms of *Porphyromonas gingivalis* – strategies used in a polymicrobial community in a heme-limited host environment. Mol Oral Microbiol 32, 1–23. doi: 10.1111/omi.12149, PMID: 26662717

[ref45] Soliman M. H. Mohamed G. G. (2013). Cr(III), Mn(II), Fe(III), co(II), Ni(II), cu(II) and Zn(II) new complexes of 5-aminosalicylic acid: spectroscopic, thermal characterization and biological activity studies. Spectrochim. Acta A Mol. Biomol. Spectrosc. 107, 8–15. doi: 10.1016/j.saa.2013.01.021, PMID: 23416903

[ref46] Wada H. (2023). 5-Aminosalicylic acid alters the gut microbiota and altered microbiota transmitted vertically to offspring have protective effects against colitis. Sci. Rep. 13:12241. doi: 10.1038/s41598-023-39491-x, PMID: 37507482 PMC10382598

[ref47] Wang H. Wang Y. Wang B. Nie J. Yan J. Hu M. (2024). Inhibitory effect of mesalazine on pro-inflammatory factors and peroxides in RAW264. 7 cells and its therapeutic effect on periodontitis model rats. J. Jilin University (Med. Edition) 50, 1250–1258. doi: 10.13481/j.1671-587X.20240508

[ref48] Xu X. Gu Z. Chen X. Shi C. Liu C. Liu M.. (2019). An injectable and thermosensitive hydrogel: promoting periodontal regeneration by controlled-release of aspirin and erythropoietin. Acta Biomater. 86, 235–246. doi: 10.1016/j.actbio.2019.01.001, PMID: 30611793

[ref49] Xue L. Y. Ouyang Q. Zhou X. G. Huang Z. H. Chen W. Chen M.. (2013). Bacterial immune interaction in experimental colitis. J. Dig. Dis. 14, 526–535. doi: 10.1111/1751-2980.12079, PMID: 23734583

[ref50] Yuan X. Zhou F. Wang H. Xu X. Xu S. Zhang C.. (2023). Systemic antibiotics increase microbiota pathogenicity and oral bone loss. Int. J. Oral Sci. 15:4. doi: 10.1038/s41368-022-00212-1, PMID: 36631439 PMC9834248

[ref51] Zhou W. Zhang X. Zhu C.-L. He Z.-Y. Liang J.-P. Song Z.-C. (2016). Melatonin receptor agonists as the “Perioceutics” agents for periodontal disease through modulation of *Porphyromonas gingivalis* virulence and inflammatory response. PLoS One 11:e0166442. doi: 10.1371/journal.pone.0166442, PMID: 27832188 PMC5104381

