# Correction: Mesalazine: a novel therapeutic agent for periodontitis via regulation of periodontal microbiota and inhibiting *Porphyromonas gingivalis*

**DOI:** 10.3389/fmicb.2025.1638611

**Published:** 2025-07-11

**Authors:** Frontiers Production Office

**Affiliations:** Frontiers Media SA, Lausanne, Switzerland

**Keywords:** periodontitis, mesalazine, *Porphyromonas gingivalis*, plaque biofilm, inflammatory bowel disease

In the published article, there was an error in Figures 2E, 2H, 4E as published. The micrometers on the scale were erroneously written as nanometers. The corrected Figures 2E, 2H, 4E, and their respective captions, appear below.

**Figure 2:**
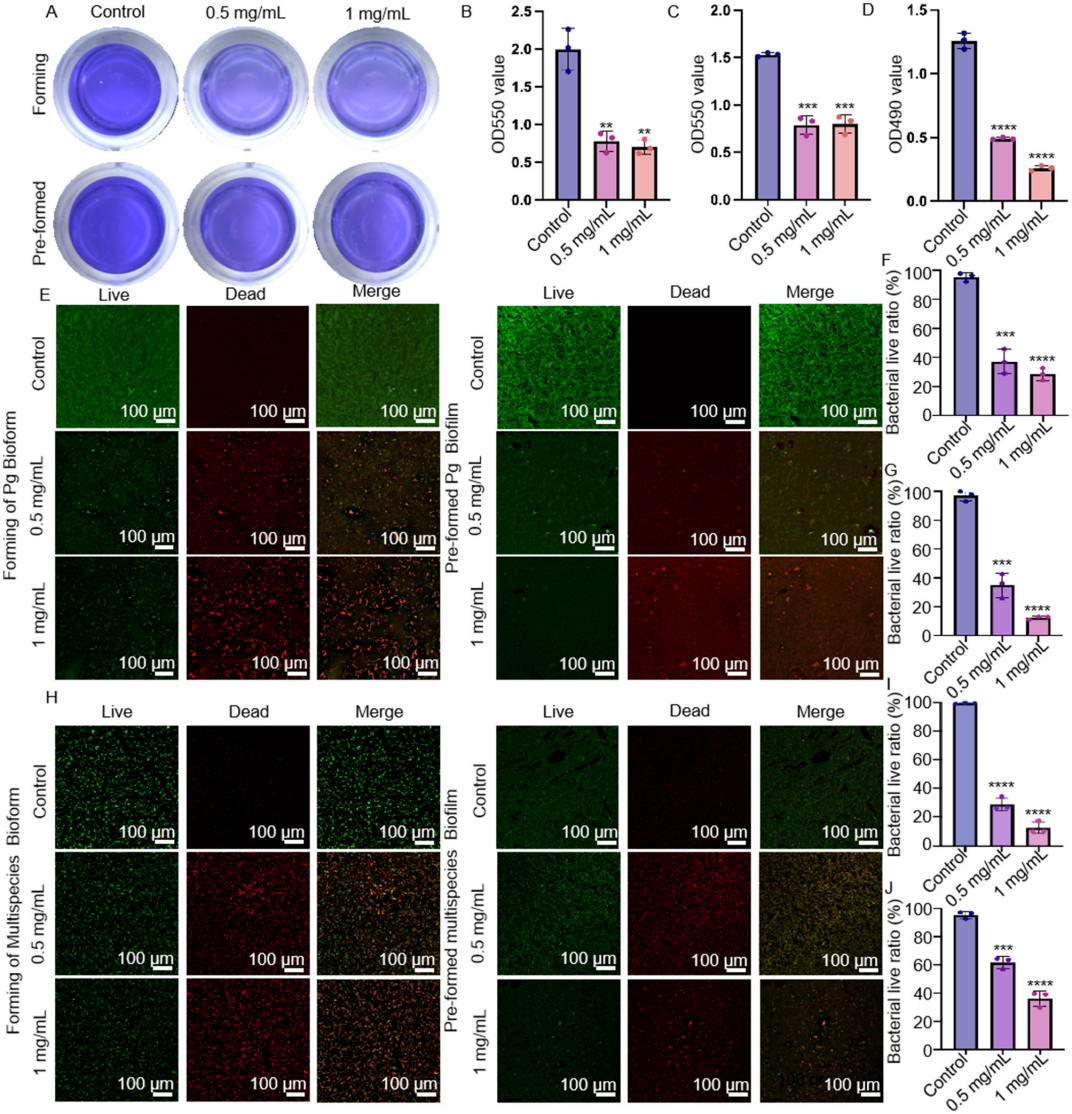
Preventive anti-biofilm potential of MSZ. **(A)** Effect of 0.5 mg/mL and 1 mg/mL MSZ on the overall biomass of *P. gingivalis* plaque biofilm formation and established plaque biofilms. **(B)** Histograms of the overall biomass of *P. gingivalis* plaque biofilm formation. **(C)** Histograms depicting total biomass distribution of pre-formed plaque biofilm spread. **(D)** Metabolic activity during plaque biofilm formation and **(E)** CLSM images of *P. gingivalis* plaque biofilm formation, including pre-formed plaque biofilms treated with varying MSZ concentrations. **(F)** Live bacteria ratio for *P. gingivalis* during plaque biofilm formation. **(G)** Live bacteria ratio for *P. gingivalis* in established plaque biofilm. **(H)** CLSM images depicting multispecies plaque biofilm formation and the effects of varying MSZ concentrations on pre-formed plaque biofilms. **(I)** Live bacteria ratio of multispecies plaque biofilm formation. **(J)** Live bacteria ratio of pre-formed multispecies plaque biofilm. Bars marked with (^**^), (^***^), and (^****^) represent significant differences at *p* < 0.01, *p* < 0.001, and *p* < 0.0001, respectively.

**Figure 4:**
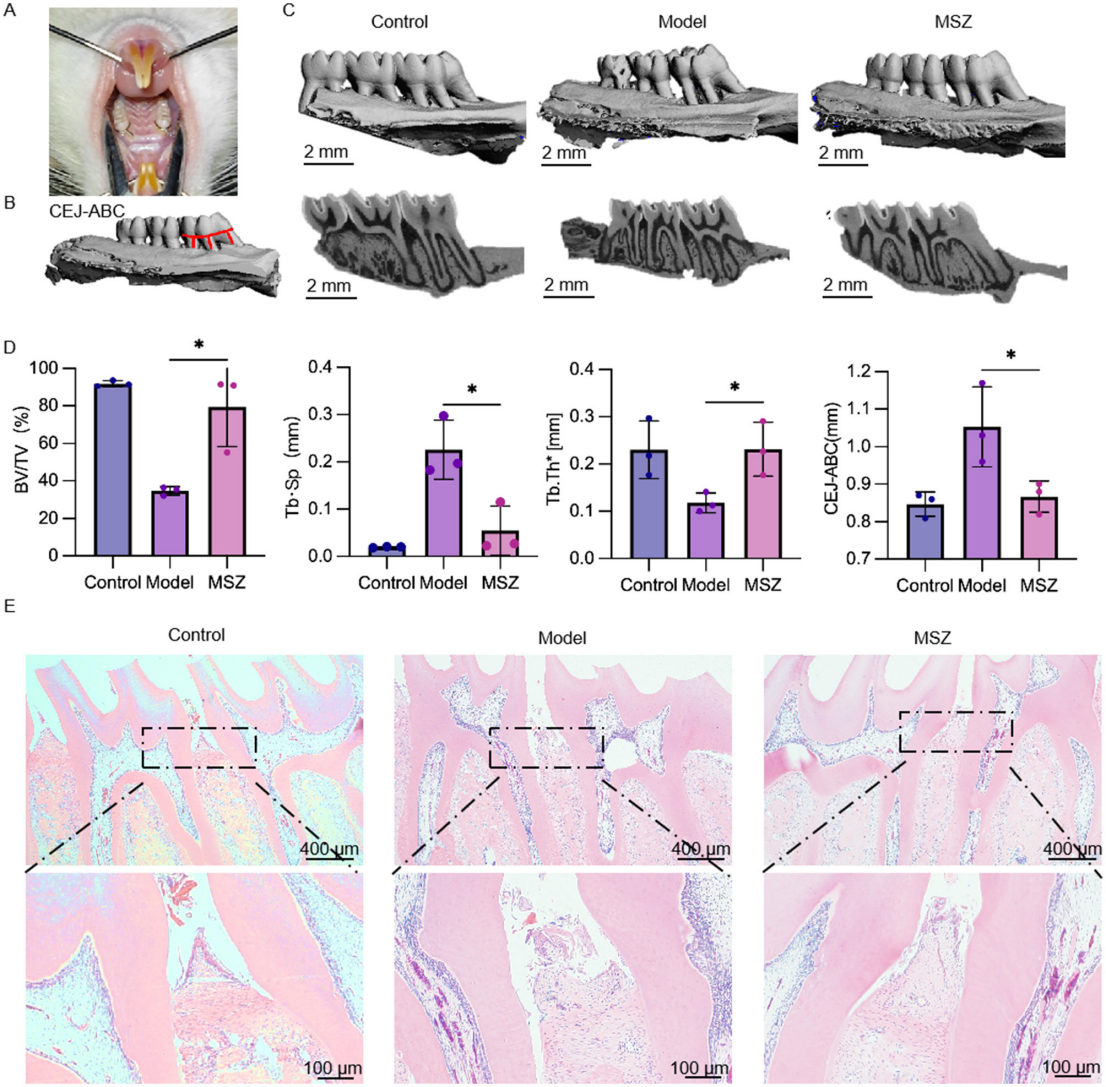
Effect of MSZ on the bone structure of the maxillary first molar in periodontitis rats. **(A)** Image of a periodontitis model. **(B)** Alveolar bone resorption of the maxillary first molar. The red line marks the CEJ-ABC distance. **(C)** Three-dimensional reconstruction and a sagittal micro-CT section of the maxillary first molar of rats in each group. **(D)** Micro-CT was used to analyze the bone structure parameters including BV/TV, Tb. Th, and Tb. Sp. The bar chart shows the CEJ-ABC distance. **(E)** H&E stained images of the periodontium were taken after 2 weeks post-treatment. First row of images (4×). Bars marked with (^*^) show a significant difference at *p* < 0.05.

The original version of this article has been updated.

